# A lightweight zero-trust authentication architecture for IoT via unified enhanced FAST-SM9 and dynamic re-authentication

**DOI:** 10.1371/journal.pone.0332943

**Published:** 2025-10-27

**Authors:** Zhanfei Ma, Hui Wei, Jing Jiang, Bisheng Wang, Hefei Wang, Zhong Di

**Affiliations:** 1 School of Information Science and Technology, Baotou Teachers’ College, Baotou, Inner Mongolia, China,; 2 School of Digital and Intelligence Industry, Inner Mongolia University of Science and Technology, Baotou, Inner Mongolia, China; 3 School of Information Engineering, Ordos Vocational College, Ordos, Inner Mongolia, China; National Sun Yat Sen University, TAIWAN

## Abstract

Authentication is a crucial challenge for Internet of Things (IoT) security, especially in open, distributed and resource-constrained environments. Current methods have significant shortcomings in terms of efficiency, adaptability, and ability to cope with complicated security threats. Therefore, this paper proposes a lightweight authentication framework for Cloud-Edge-End, which integrates the enhanced Fast Authentication and Signature Trust for SM9 (FAST-SM9) algorithm and zero-trust Dynamic Re-authentication (zero-trust-DRA) mechanism. First, FAST-SM9 effectively reduces protocol overhead, and meanwhile ensuring security by organically integrating authentication and signature processes. Its architectural optimization reduces the number of communication rounds by 40% and simplifies trust negotiation between heterogeneous layers without affecting the integrity of encryption mechanisms. To enhance runtime protection, the designed zero-trust-DRA mechanism also introduces context-aware, time-windowed based re-authentication techniques so as to efficiently defend against risks such as session hijacking and credential leakage. In addition, the Dynamic Identity Token Generation Mechanism (DITGM) enhances the security and flexibility of the system by incorporating multi-factor attributes such as fingerprints and OTP seeds into time-sensitive tokens. Experimental results show that this scheme reduces latency by 56.6% and energy consumption by 63% compared to traditional PKI edge authentication methods, and effectively resists related attacks. The formal tool AVISPA verification further confirms its security. The scalability testing also proves its applicability in IoT. A feasible path is provided for efficient and secure identity authentication in distributed systems, which helps to promote the development of zero-trust security systems.

## 1 Introduction

The Internet of Things (IoT) is significantly driving growth in all areas and is expected to connect huge numbers of devices, creating enormous economic value [1]. However, the rapid growth in the number of devices has exacerbated security challenges, particularly in authentication. As a critical aspect of IoT systems, authentication is vulnerable to attacks. Traditional schemes such as PKI and OTP suffer from computational complexity, centralized bottlenecks, and static trust assumptions, which can easily lead to replay attacks, credential leakage, and even quantum security risks [2–4]. For example, OTP-based smart homes often suffer from authentication vulnerabilities, and there may cause unacceptable real-time delays [5,6] in PKI applications in industrial IoT.

The SM9 standard, as the latest development in identity-based cryptography, eliminates certificate management and achieves lightweight and quantum-resistant authentication [7], providing a promising alternatives. Although it has significant advantages, existing SM9 implementations typically require multi-step interactions, which may affect efficiency in dynamic environments [8]. Meanwhile, although the zero-trust architecture stresses continuous verification, it has not yet been effectively integrated with encryption primitives designed specifically for restricted environments of the IoT [9]. This disconnect reveals a key research gap. There exists a lack of a unified elastic framework that can integrate efficient cryptographic mechanisms, dynamic trust models, and distributed IoT ecosystems [10,11].

To address these challenges, this paper proposes a new authentication framework for Cloud-Edge-End, integrating the strengths of SM9 and dynamic, context-aware re-authentication strategies. The key design is Fast Authentication and Signature Trust for SM9 (FAST-SM9). It is a refined, lightweight extension of the SM9 algorithm. Unlike existing implementations, the identity verification and digital signature generation are integrated into a unified cryptographic process in FAST-SM9. This integration reduces communication overhead and processing latency by more than 40% and enhances operational resilience for real-time, low-power IoT environments.

This study also proposes a practical technical path for constructing an authentication architecture with both quantum-resistant and context-driven features, which can further promote the evolution and development of zero-trust security systems in distributed systems. The quantum resilience of our scheme comes from the FAST-SM9 algorithm, which is based on the SM9 standard and utilizes Elliptic Curve Cryptography (ECC) with large key sizes and bilinear pairings. Compared with traditional RSA-based systems, these cryptographic primitives have a higher security margin against quantum attacks, because breaking them using quantum algorithms such as the Shor’s algorithm requires more computational resources.

Furthermore, we propose a zero-trust Dynamic Re-Authentication (zero-trust-DRA) mechanism that uses session-aware and time-window-based triggers to implement adaptive identity reassessment, thus preventing long-term credential abuse and session hijacking.

To further improve the system security, we propose a Dynamic Identity Token Generation Mechanism (DITGM) that embeds real-time attributes such as device fingerprints, OTP seeds, and contextual metadata into time-sensitive tokens. Thus it achieves flexible multi-factor authentication and excellent token freshness assurance. The designed Failure Management Mechanism (FMM) can identify and respond to abnormal behaviors, such as repeated identity verification failures, through device locking and alert notifications. Based on this, FMM mitigates the security threats that are caused by brute force attacks.

This study is the first to integrate the FAST-SM9 algorithm with zero-trust-DRA and DITGM. It applies SM9 to zero-trust thinking to establish a cohesive and scalable architecture for heterogeneous and large-scale IoT environments. This framework supports distributed authentication across Cloud-Edge-End layers. This ensures low-latency authentication, and global trust coordination. Our approach significantly improves scalability, responsiveness, and resilience compared to static centralized systems.

In summary, our main contributions are as follows:

(1) We propose an innovative scheme based on the FAST-SM9 algorithm and zero-trust-DRA mechanism. This scheme addresses the shortcomings in current research regarding the authentication process, poor security, and single points of failure. Our solution offers a more practical approach, achieving a better balance and optimizing these issues. Specifically, we introduce a novel protocol design that unifies authentication and signature in a one-pass operation via the ISP structure, reducing communication rounds by 40% while maintaining SM9’s bilinear pairing security under a formal Bellare-Rogaway model (detailed in Section 4.6).
(2) We propose an enhanced FAST-SM9 algorithm that innovatively integrates authentication and digital signature verification into a unified operational process by introducing an Improved Single Package (ISP) structure into SM9. This structure implements a one-time processing of “authentication+signature”, which not only ensures the authenticity of identity and the integrity of data, but also effectively reduces the number of encryption operations and communication interaction rounds in the SM9 algorithm. Compared to traditional solutions, this method significantly improves the throughput of authentication, reduces computation and communication latency, and is particularly suitable for resource constrained IoT devices, with stronger advantages in lightweight and efficiency. The architectural modeling emphasizes state transitions and modular interactions across Cloud-Edge-End layers, as formalized in Section 3.
(3) We propose a zero-trust Dynamic Re-Authentication (zero-trust-DRA) mechanism that dynamically initiates re-authentication using context-aware, multi-dimensional triggering conditions to enhance session security within the zero-trust architecture. This mechanism flexibly adapts to changes in environment and behavior by persistently assessing session validity based on factors such as time-window expiration, user inactivity thresholds, device location changes, access patterns, network context, and sensitive operation requests. The zero-trust-DRA mechanism achieves fine-grained adaptive access control by re-evaluating access requests in real-time without affecting user experience, which effectively reduces security risks such as session hijacking and long-term credential abuse.
(4) We develop a DITGM that fuses device-centric features such as hardware fingerprints, OTP seeds, and real-time context into non-reusable, attribute-bound authentication tokens. These tokens are cryptographically time-bound and can support multi-identity or multi-level access scenarios, enabling the system to flexibly switch between different authentication modalities while preserving both security and efficiency.

In most performance and security comparison experiments, our scheme outperforms existing research results in most cases. The remainder of this paper is organized as follows: Section 2 reviews related work and highlights the limitations of existing authentication models. Section 3 details the proposed framework. Section 4 analyzes its security properties. Section 5 presents experimental evaluations. Finally, Section 6 concludes and outlines future research directions.

## 2 Related works

### 2.1 Zero-trust architecture

Zero-Trust Architecture (ZTA) was originally designed to protect internal corporate networks [12]. Traditional ZTA security models typically use perimeter-based defenses that implicitly trust devices and users once they access the internal network [13]. However, with the increasingly distributed and dynamic nature of modern computing environments, these models are facing more and more challenges. Existing research on ZTA [14–16] mostly depends on centralized authentication methods, which often result in questions such as authentication delays and single-point-of-failure vulnerabilities in Cloud-Edge-End architectures, thus weakening the overall resilience of the system.

To overcome the above limitations, researchers have recently begun to explore decentralized authentication frameworks [17–19], context-aware dynamic access control [20,21], and intelligent decision-making measures. They want to improve the adaptability and utility of ZTA when faced with real-world security threats. However, existing research (e.g., literature [22]) tends to focus on superficial integration of the zero-trust concept and lacks key mechanisms such as session re-authentication and time-window based authentication, which are critical for maintaining continuous trust. This absence expands the attack aspects of the system. Especially during long sessions, it increases the risk of credential leakage and session hijacking [23].

Moreover, prevailing approaches exhibit a high trust assumption for internal entities, which makes them susceptible to internal threats such as malicious insiders or credential leakage. At the same time, their protection against external, complex, and heterogeneous attack vectors remains insufficiently defined [24].

To solve the above problems, we propose the zero-trust-DRA framework that is an enhanced scheme, which integrates dynamic re-authentication with a time-window based verification mechanism. The framework achieves fine-grained trust assessment over the full lifecycle of a session by combining FMM with a context-aware adaptive policy. Meanwhile, continuous behavioral monitoring and periodic authentication efficiently prevent external threats such as session hijacking and identity impersonation. It is worth emphasizing that the scheme integrates an enhanced FAST-SM9, which can seamlessly integrate the authentication and digital signature processes, significantly improves the efficiency of trust verification in dynamic distributed environments, and meanwhile reduces the number of communication rounds and computational burden.

### 2.2 Authentication based on OTP

The rapid development of e-commerce and online service platforms has driven the widespread use of One-Time Password (OTP) mechanisms in areas such as banking [25], online payments, and social networks [26]. Meanwhile, OTP has evolved from a simple authentication method to become a crucial part in protecting sensitive operations in multi-factor authentication frameworks.

Although traditional OTP schemes has advantages for instant authentication, they are inherently designed for one-time authentication [27], which makes them unsuitable for environments that requires continuous trust assurance during ongoing sessions or frequent device interactions. The existing authentication mechanisms are often designed as a binary decision-making process, where once a user is verified, the system defaults to continuing to trust the user throughout the entire session [28]. For instance, although [29] proposed a protocol combining a decentralized microservice architecture and blockchain-based OTP framework (MBB-OTP), the system are vulnerable to identity forgery and unauthorized persistent access because of its high reliance on OTP. Similarly, although [25] simplifies the two-step verification into a single OTP stage to improve usability, this scheme exposes the system to potential attacks once the OTP is intercepted. Despite the introduction of a timed OTP mechanism based on chaotic mapping in [30], there still exists a lack of session state tracking and mid-session identity re-validation.

To solve these limitations, we design a new scheme that called DITGM. OTP serves as an auxiliary credential rather than the sole authentication factor in this scheme. This design effectively alleviates excessive reliance on OTP. Even if OTP is leaked, it will not cause unconditional access permission being granted. Meanwhile, this method only introduces minimal communication overhead, and brings significant improvements in security and robustness. In addition, we have constructed a comprehensive, real-time, and resilient security system by integrating the dual authentication strategy of OTP and zero-trust-DRA, which combines with session aware dynamic re-authentication and time-window based credential verification. No longer limited to one-time verification, but continues to provide dynamic trust assurance throughout the session lifecycle.

### 2.3 SM9 algorithm and sing-package mechanism

SM9 algorithm is an identity-based cryptographic mechanism [31] that can directly generate public-private key pairs using the identity attributes of user (e.g., user ID or device MAC address), thus avoiding the complex processes of certificate issuance, verification, and lifecycle management in traditional cryptographic systems. SM9 demonstrates significant advantages in simplifying the authentication process and improving the signature efficiency compared with the traditional PKI-based encryption system [32].

Recent research has begun to explore the combination of SM9 with Single-Package authorization mechanisms to achieve continuous authentication in dynamic environments. However, there is still a lack of in-depth exploration in existing research on the systematic and efficient integration of these two aspects [33–35]. For example, [36] employs a self-designed security framework that integrates single-package authorization into a zero-trust system, but it still suffers from multi-step interaction overhead and sub-optimal efficiency, restricting its applicability in latency-sensitive scenarios. Similarly, literature [37] combines the lightweight encryption primitive ASCON with elliptic curve cryptography for constructing a resource-friendly and secure communication channel. Although this scheme enhances data protection ability between edge devices, it still focuses on the optimization of encryption performance and lacks a systematic exploration of mechanisms such as authentication, dynamic access control, and persistent trust evaluation in zero-trust architectures. This disadvantage makes it difficult to meet the demand for adaptive security policy in dynamic heterogeneous environments.

In contrast, we proposes an Improved Single-Package (ISP) authorization mechanism. It consolidates identity information and OTP seeds into a unified transmission unit, achieving a more efficient integrated authentication process. This mechanism simplifies the authentication process, reduces communication epochs and maintains necessary security. We integrated it with SM9 without relying on certificate-related operations, further optimizing the overall process and enhancing its adaptability in heterogeneous and dynamic application environments. [38] proposes an efficient SM9 aggregate signature scheme to reduce verification overhead, but it fails to integrate with a continuous authorization framework and does not involve session persistence [24,39,40] or the design of adaptive authentication policies.

Given the above drawbacks, we design a dynamic identity authentication scheme that integrates FAST-SM9, zero-trust-DRA and DITGM in a unified framework. This scheme achieves lightweight and maintains high robustness. It can balance algorithm performance and adaptive trust evaluation capability. By supporting multi-attribute identity validation, fast trust update mechanism, and effective defense against complex attacks such as identity forgery and session hijacking, this scheme provides a highly secure architecture to solve the complexity of next-generation network systems.

## 3 Methodology

**Fig 1:**
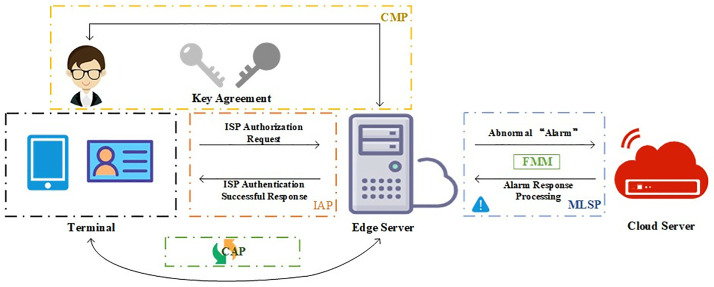
The overall identity authentication flowchart based on FAST-SM9 algorithm and zero-trust-DRA mechanism.

The identity authentication scheme we propose is shown in Fig 1. It consists of an initial authentication phase (IAP), a continuous authentication phase (CAP), a failure management mechanism (FMM), multiple layers of security policies (MLSP), and a communication maintenance phase (CMP). Among them, the IAP stage and CMP stage together constitute our proposed FAST-SM9 algorithm. Together, the two mechanisms in the CAP and CMP stages constitute zero-trust-DRA mechanism. The relevant symbols and their meanings are shown in Table 1.

**Table 1:** Symbols and descriptions.

Symbols	Descriptions
*PK*	The master public key
*SK*	The master private key
*Da*	Terminal private key generated via elliptic curve operations
*OTP*	One-Time Password
*OTP_Seed*	Random seed for generating OTP (a high-quality 16-byte random number encoded)
*Identity*	Unique identifier for a user or device
*Fingerprint*	Device-specific attributes (e.g., MAC address, serial number)
*HardwareInfo*	Hardware feature information
*Counter*	Tracks the number of authentication attempts
*HMAC*	Hash-based Message Authentication Code
$H_{1}$	Cryptographic hash function used in SM9
*H2RF*	Hash-to-Random Function for challenge value generation
*q*	Order of the elliptic curve
$P_{1}$	Public key point on the elliptic curve
*m*	Challenge value for private key generation
*e*	Bilinear mapping tool for session key computation
KEM	Key Encapsulation Mechanism
DEM	Data Encapsulation Mechanism
$P_{pub}$	The main public key generated by PKG
*s*	The main private key generated by PKG
*P*	Base point on the elliptic curve
$D_{A}$	Extract the private key of the terminal
$D_{B}$	Extract the private key of the server
*hid*	Key type identifier
*Fail_Logs*	Logs of failed authentication attempts
*Error_Counts*	Record of authentication failure details
*r*	The order of the elliptic curve group (the size of the finite field)
*t*	A shared key calculated based on bilinear pairings

### 3.0 System architecture overview

Before delving into the phases, we provide an overview of the system hierarchy to clarify module boundaries and interactions. The framework operates in a Cloud-Edge-End distributed model: End devices initiate authentication and handle lightweight computations; Edge servers manage real-time token generation (DITGM) and dynamic triggers (zero-trust-DRA); Cloud oversees global trust coordination (FMM for locking/escalation). Modules are non-overlapping: DITGM generates time-bound tokens with multi-factors (e.g., fingerprints, OTP); zero-trust-DRA triggers re-authentication via context (e.g., time-windows > 5s inactivity, location shifts); FMM escalates failures (e.g., > 3 errors lock device). Interactions follow a state machine: IAP → CAP (if success)→CMP; failures route to FMM. Performance distribution: Edge absorbs 70% latency for local authentication; Cloud 20% for audits; End 10% for signatures. Boundary security uses FAST-SM9 encrypted channels (e.g., bilinear pairings for inter-layer key exchange).

### 3.1 Initial authentication phase

In the initial authentication stage, we use the ISP authorization mechanism combined with multi-attribute key generation based on the FAST-SM9 algorithm. First, the terminal sends the relative message to the edge server to generate a unique identifier. This identifier will provide a basis for other authentication information (such as *OTP*, *Timestamp*, etc.). *Timestamp* is the current verification request time. We uniquely combine identity information with an *OTP_ Seed* as the first input parameter and use a *Counter* as the second input parameter. Then, the HMAC algorithm is used to process them and obtain the *OTP*:


Message=(Identity || Fingerprint || HardwareInfo || Timestamp) (1)

Among them, *Identity* is the user’s identity identifier, *Fingerprint* represents fingerprint information (which can be MAC address, hardware serial number, etc., in real environments), and *HardwareInfo* is the hardware’s feature information. These sent messages will be updated with each authentication request to prevent Replay Attacks. The edge server will generate the user’s *Identity* and *OTP_Seed* based on the received information. The corresponding *Identity* identifier is then stored in association with the *OTP_Seed*.

Then, after receiving the terminal information, the edge server uses the SM3 Hash function $H_{1}$ to generate a unique *Identity* identifier based on the relevant information of the terminal:


$$Identity=H_{1}(Fingerprint\;||\;HardwareInfo)$$ (2)

This *Identity* identifier will be used as the core basis in the subsequent authentication process to ensure the uniqueness for each terminal device. The edge server will also generate an *OTP* for dynamic identity token authentication based on this information.

After receiving the *Identity* identifier transmitted by the server, the terminal begins to generate a private key based on the generated identity information, as well as the *PK* and *SK*, and uses the *PK* to sign the authentication message. The key steps are as follows:

Firstly, the *Identity* is converted into a byte string and hashed by using the hash function $H_{1}$ to generate the user’s hash value *user_id*. The identity identifier is hashed to ensure consistency and privacy of the identity information:


$$user\_id=H_{1}(Identity)$$ (3)

Then, use the H2RF function to generate a challenge value *m* based on the *user_id*, and ensure that it is a valid value within the curve order (*ec.curve_order*) range, which can avoid calculation overflow or invalid input, and ensures that the mathematical operations are secure and correct. The randomness and unpredictability of *m* ensure that the protocol is secure and prevents attackers from forging or predicting the challenge value, thus enhancing the security. H2RF is a randomized function based on a hash function that generates a suitable value within curve order range using *user_id* and constant inputs:


$$m=H2RF(user\_id\;|{|}^{\prime }01^{\prime },ec.curve\_order)$$ (4)

Afterwards, we add the *SK* to the generated challenge value *m* and check if the result is zero. Otherwise, the generated value *m* is considered invalid. A failure then is returned:


m=SK+m (5)

The purpose of this step is to prevent *m* from entering an invalid range. During the calculation process, once *m* = 0 or involves multiplication with zero, it may result in the disappearance of multiplication factor, rendering the encryption or signature formula invalid. Therefore, by regenerating *m* to ensure its randomness, it not only improves the system’s defense against various attacks, but also effectively avoids weak key issues that may arise in specific scenarios.

Moreover, we use the *q.prime_field_inv*() (possibly an inverse operation function) and *SK* multiplication to obtain a valid and effective private key m for the next step. *q.prime_field_inv*() function is used to ensure the validity of *m* in a finite field:


m=SK×q.prime_field_inv(m,ec.curve_order) (6)

Finally, we choose the public key point *P*1 and obtain the final private key *Da* through elliptic curve multiplication *ec.multiply*. *P*1 is a coordinate pair on an elliptic curve:


Da=ec.multiply(P1,m) (7)

The multiplication here is not a traditional numerical multiplication, but refers to point multiplication on elliptic curves. It follows the geometric rules defined by curve equation. The symbol *Da* that is obtained by scalar multiplication denotes a new point on the elliptic curve, which plays a critical role in the public key generation process as part of the private key.

After the above process, the terminal uses the SM9 algorithm to sign the *Identity*, *Da*, and message *msg* to obtain the *Signature*:


Signature=Sign(PK,Da,msg) (8)

The *msg* contains authentication messages, and the Sign is a signature function.

Finally, after generating the signature, the terminal sends both the authentication message and the signature to the edge server for verification. After receiving the message, the cloud server verifies the validity of the signature using the corresponding *PK* to ensure that the authentication message transmitted during the ISP authorization process has not been tampered with, and confirms that the message was indeed sent by the legitimate user. This approach improves authentication efficiency while ensuring the integrity of the authentication process and greatly reducing the risk of intermediate steps being tampered with:


Verify(PK,(msg,Signature))⇒Ture / False (9)

The core concept of FAST-SM9 is reflected in this step, which fuses authentication and digital signature generation into a unified cryptographic operation. By innovatively merging these steps, FAST-SM9 efficiently reduces computation and communication iterations, which ensures both authentication and data integrity with just one operation. Compared to the existing SM9 algorithm and Single-Package authorization mechanism, FAST-SM9 significantly improves the speed and security of authentication by simplifying the calculation steps and reducing communication overhead. This optimization advances system performance while embodying the unique innovation of security protocol design.

### 3.2 Continuous authentication phase

After successfully verifying the signature, the system enters the dynamic identity token continuous authentication phase. At this stage, the edge servers utilized the generated *OTP_Seed* and *Identity* while combining current *Timestamp*, generating a Dynamic Identity Token (*DIT*), which is then verified by the terminal. This token is updated with each authentication request and is designed based on time constraints to efficiently prevent Replay Attacks and Identity Forgery:


DIT=HMAC(OTP_Seed || Identity || Timestamp) (10)

In the DITGM scheme, *DIT* is time-bound to ensure freshness and prevent reuse. The default expiration time for each token can be adjusted according to the security requirements of the system (e.g., shorter for high-sensitivity operations). Once the token expires, it cannot be used anymore and a new token needs to be generated for the next authentication request. Token revocation will be triggered in two situations: (1) if abnormal behavior is detected, such as multiple consecutive authentication failures exceeding a threshold, the edge server will add the token to the cloud server’s lock list; (2) The user or administrator voluntarily ends the session. The edge server communicates with the cloud server to update the token status and ensure that revoked tokens cannot continue to be used for access. See Section 3.3 for details. This method enhances security by limiting the opportunity window for attackers to exploit stolen tokens.

The zero-trust-DRA mechanism introduced in this section mainly achieves dynamically triggered identity re-authentication through context-aware parameters. Specially, these parameters include device location information (e.g., inferred from IP addresses) and access behavior patterns (e.g., frequency of authentication requests, time distribution, and reasonable authentication time window). These factors can flexibly trigger the re-authentication process based on environmental changes or different user behaviors. This mechanism can enhances security without interfering with user experience (Details are presented in 3.4). In future research, we plan to introduce more context-aware parameters that are more realistic to simulate the operational requirements in different application environments. Throughout the entire session, this method also periodically performs authentication to ensure that each request is validated based on a valid dynamic identity token, which prevents the session from being hijacked:


Valid=Verify(OTP,Identity,Timestamp) (11)

Compared with existing static password and *OTP* authentication methods, we propose DITGM that introduces a dynamic continuous authentication mechanism. It can adjust based on real-time changing authentication information and ensure the effectiveness of authentication through continuous verification. This strategy implements the core idea of zero-trust-DRA, which is that both internal and external entities should not default to trust (this step, together with the subsequent window authentication mechanism, constitutes the zero-trust-DRA mechanism). The real-time authentication-based approach effectively prevents Replay Attack and radically reduces the risk of attackers using stolen and outdated authentication information for forgery or counterfeiting.

### 3.3 Failure management mechanism and multi-layers security strategy

We propose a FMM and a multi-layer security strategy to enhance the fault tolerance and security of the system, and provide a referenceable fault handling mechanism. When an authentication request fails (i.e., a dynamic identity token authentication error), the system will accumulate the *Counter* once. When the *Counter* value reaches the set threshold (which can be flexibly configured according to actual needs), the system will automatically trigger the corresponding alarm mechanism.

The failure counter will increase after each authentication request. If the authentication request is successful, the counter will be reset to zero. This method can reduce the false alarm rate of legitimate devices.

Then, if the failure counter reaches the set threshold, the edge server first sends the *Identity* and lock request of the violating terminal to the cloud server. The cloud server obtains this request data, extracts the terminal identity, adds the terminal to the lock list, and returns a successful lock message to the edge server. The edge server locks successfully.

**Fig 2:**
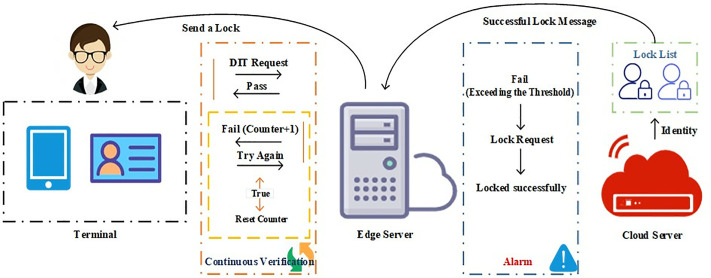
FMM and multi-layer security strategy diagram.

The terminal cannot initiate new requests during the period marked as locked. After receiving a lock request, the cloud server will store the lock information of the terminal, as shown in Fig 2, and block its request for a certain period. When the lock time expires or the administrator manually unlocks, the cloud server will be unlocked.

Besides, this method also records every failed authentication attempt *Fail_Logs* for later analysis and auditing. All failed attempts *Error_Counts* include the following information: hashed identity, authentication failure type *Error_Type* (such as dynamic identity token error, input format error, etc.):


$$Fail\_logs=(Identity(H_{1}(Identity)),Error\_Type,Error\_Counts)$$ (12)

On balance, our FMM is responsible for handling faults and errors. In the layered architecture of Cloud-edge-end, as the executor of multi-layer security policies, they implement corresponding handling measures respectively. Meanwhile, analyzing and documenting failures is also an important part of multi-layered security strategies, which helps to enhance the stability and security of the system. When the cumulative number of identity authentication failures exceeds the preset threshold, the mechanism will automatically initiate a series of measures that include timed retries and detailed recording of failure details. These measures comprehensively enhance the system’s fault tolerance and overall security. Meanwhile, once authentication is successful, the system will immediately reset the failure counter, which can minimize the misjudgment rate of legitimate devices.

### 3.4 Maintain communication session phase

After the ISP authorization mechanism and dynamic identity token authentication in FAST-SM9 are successful, the FAST-SM9 key is used for negotiation to establish a communication session and exchange messages.

Firstly, PKG generates the master public key $P_{pub}$ and master private key *s*, where *s* is a randomly selected large integer and *P* is the base point on the elliptic curve. In a decentralized key management scheme, PKG provides key generation services. At this point, PKG distributes the $P_{pub}$ for subsequent key negotiations, while *s* is kept confidential:


master_secret=s (13)

$$master\_public=P_{pub}=s\cdot P$$ (14)

**Fig 3:**
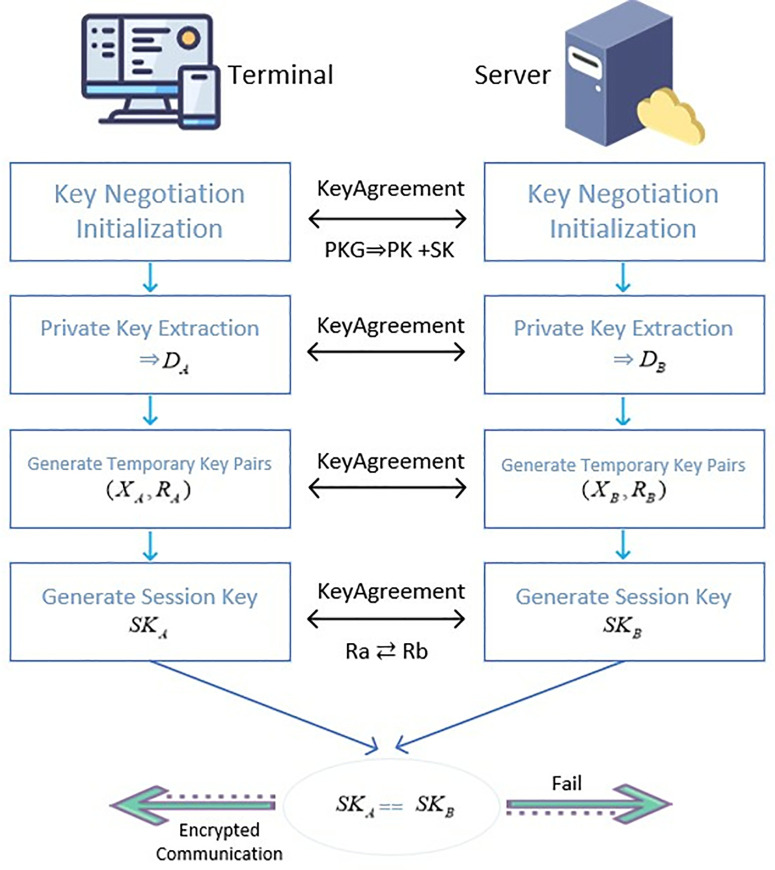
Key negotiation and session establishment diagram.


The terminal and server respectively request the private key from PKG, as shown in Fig 3. Then, the terminal and server obtain private keys separately:

$$D_{A}=s\cdot H_{1}(ID_{A}\;||\;hid,q)$$ (15)

$$D_{B}=s\cdot H_{1}(ID_{B}\;||\;hid,q)$$ (16)

At this point, $ID_{A}$ and $ID_{B}$ are the identifiers of the terminal and server respectively.

Next, the terminal and server generate a temporary key pair (X,R), respectively, which $X_{A},X_{B}$ are random numbers (they are integers between 0 and *r*-1), and $R_{A},R_{B}$ are the temporary public keys of the terminal and server, used for exchanging information:


$$X_{A}\in Z_{r}^{*},R_{A}=X_{A}\cdot P$$ (17)

$$X_{B}\in Z_{r}^{*},R_{B}=X_{B}\cdot P$$ (18)

Then, the terminal and server calculate the shared session key separately. The terminal and server calculate their respective medians $g_{A},g_{B}$ using their private key and the other party’s temporary public key:


$$g_{A}=e(D_{A},R_{B})$$ (19)

$$g_{B}=e(D_{B},R_{A})$$ (20)

This median is fused through *e* and ultimately utilized to calculate the shared session key to ensure secure data exchange between communication parties and maintain the confidentiality and integrity of the encrypted channel. Then, compress the result into a shared session key using $H_{1}$:


$$SK_{share}=H_{1}(g^{X_{A}\cdot X_{B}},ID_{A}\;||\;ID_{B}\;||\;R_{A}\;||\;R_{B})$$ (21)

Finally, by utilizing the properties of bilinear pairs $e(aP,bQ)=e(P,Q{)}^{ab}$, the consistency of the session keys $g_{A},g_{B}$ generated by the terminal and server are verified, thereby ensuring the security of the session keys. Among them, *aP* and *bQ* are scalar multiples of points *P* and *Q*, respectively, where *P* and *Q* are two points on the elliptic curve. *a* and *b* are integers (usually randomly selected private keys representing a part of authentication). *e*(*P*, *Q*) is the result of bilinear mapping between *P* and *Q.* $e(P,Q{)}^{ab}$ means encrypting the bilinear mapping result of *P* and *Q*, and generating a shared key through the private keys (*a* and *b*) of the two parties involved. Even if PKG is not online, the terminal and server can still complete key negotiation:


$$g_{A}=g_{B}\Rightarrow SK_{A}=SK_{B}$$ (22)

During this process, PKG mainly completes the generation of master key pairs $\left(s,P_{pub}\right)$ and generates the private key based on the *Identity* (only participates in the initialization phase and does not involve subsequent session key generation).

**Fig 4:**
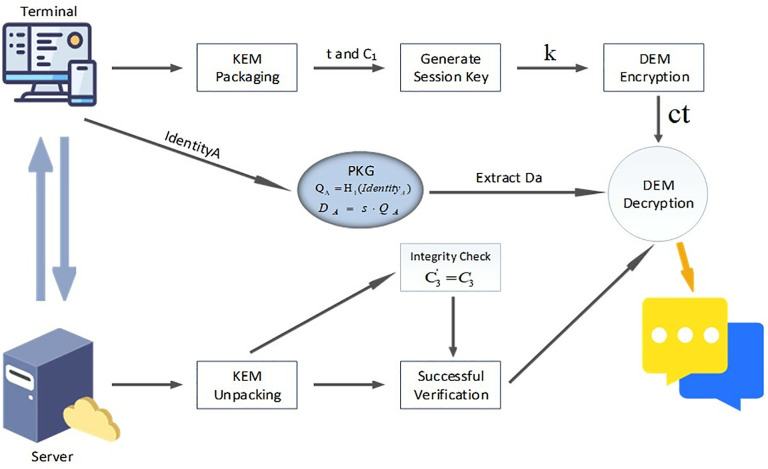
Display diagram of KEM + DEM session message processing mechanism.


After the above operations, in the message verification stage, as shown in Fig 4, the terminal extracts the private key *Da* through PKG. $Q_{A}=H_{1}(Identity_{A})$ is the elliptic curve point generated based on the identity. The PKG uses the main private key *s* to calculate the terminal’s private key $D_{A}$ and then distributes it to the terminal:

$$D_{A}=s\cdot H_{1}(Identity_{A})=s\cdot Q_{A}$$ (23)

The terminal then starts encapsulating messages and data by the KEM + DEM mechanism. KEM is responsible for secure key exchange, while DEM is responsible for data encryption and decryption:


$$X\overset{\;\$\;}{\leftarrow }\;Z_{r},C_{1}=x\cdot Q_{B},t=e(P_{pub},Q_{B}{)}^{X}$$ (24)

Among them, *X* is a randomly generated private key, $C_{1}$ is a temporary public key, and *t* is a shared key calculated based on bilinear pairings $e(P_{pub},Q_{B}{)}^{X}$.

Secondly, the session key *k* is generated as a symmetric key derived through the key derivation function KDF. Converting elliptic curve points and finite field elements into byte strings is the responsibility of EC2SP and FE2SP:


$$k=KDF(EC2SP(C_{1})\;||\;FE2SP(t)\;||\;H_{1}(Identity_{B}))$$ (25)

Then, DEM encryption is performed, in which *M*[*i*] is the *i*-th byte of the message. $k_{1},k_{2}$ are the segment of *k*. $C_{2}[i]$ is the temporary public key of the *i*-th byte:


$$C_{2}[i]=M[i]⊕k_{1}[i],i\in [1,len(M)]$$ (26)

In summary, the complete ciphertext is as follows. $C_{3}$ is a temporary public key that needs to be verified:


$$C=(C_{1},C_{2},C_{3}),C_{3}=H_{1}(C_{2}\;||\;k_{2})$$ (27)

Next, the server decrypts the message and recovers the message through the ciphertext *ct* and private key $D_{B}$ sent by the terminal, completing the communication:

1) KEM unpacks the package *t*, *k*:

$$t=e(C_{1},D_{B}),k=KEM(k)$$ (28)

2) The server performs integrity verification. $C_{3}^{\prime }$ is the identity data processed by the $H_{1}$, used for security checks:

$${C_{3}}^{\prime }=H_{1}(C_{2}\;||\;k_{2}),Verify\mathrm{\backslash nolimits}C_{3}^{\prime }=C_{3}$$ (29)

3) Finally, there is DEM decryption:

$$M[i]=C_{2}[i]⊕k_{1}[i],i\in [1,len(M)]$$ (30)

During the message interaction phase, our window authentication mechanism plays a key role and forms a complete zero-trust-DRA mechanism together with the continuous authentication mechanism in Part B. By combining these two authentication mechanisms, the system can dynamically evaluate and verify the identities of participants in each authentication cycle. This scheme ensures that both internal and external entities are not trusted by default. Thus it significantly improves the security and attack resistance of the system. We will introduce the following in detail.

If the counter is not incremented when verifying the counter, it is treated as a duplicate message and discarded. This avoids duplicate messages within the time window, so attackers cannot deceive the server through Replay Attacks.

**Fig 5:**
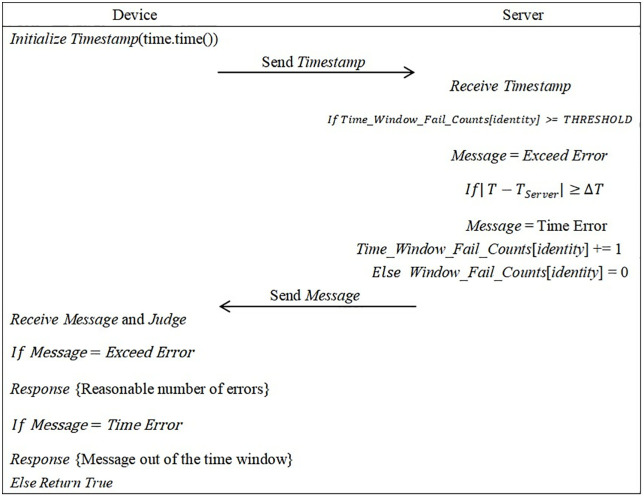
Continuous message window authentication diagram.

The experimental simulation process is shown in Fig 5. If the edge server detects that the window message exceeds the reasonable verification time, it will refuse to process the next operation. The client receives a response from the edge server and displays that the verification window has failed, and needs to wait for a retry (at this time, the timestamp data content is not visible in the real environment, and is displayed for comparison purposes). If the window fails more than a reasonable number of verification, the edge server reminds the client that abnormal behavior has been detected, but verification can still continue. At this time, the edge server also sends abnormal information to the cloud server for audit records. If within the normal message window authentication time, the window audit frequency is reset to mitigate false positive incidents for legitimate users.

## 4 Safety analysis

### 4.1 Experimental environment

Our experimental simulation is conducted under the following hardware configuration: the processor is 13th Gen Intel® Core™ i7-13700 (2.10 GHz), and the memory is 32GB. The software operating environment is Python 3.11, the main libraries used include Socket, Threading, Time and Pickle, and so on. In the experiment, the server runs on two independent ports, simulating computing nodes on the cloud and edge, respectively. The IP address of the simulation server is 127.0.0.1, and the cloud port number is 5002 (corresponding to URL http://127.0.0.1:5002). The edge port number is 5000 (corresponding to URL http://127.0.0.1:5000). Terminal interaction is achieved through another independent window.

Within a reliable time window, our experiment has successfully implemented a high-performance multi-level identity authentication method based on the FAST-SM9 algorithm and zero-trust-DRA mechanism across the entire Cloud-Edge-End architecture.

### 4.2 Penetration testing and attack simulation

To verify the resilience of the scheme in a real network environment, we design multi-dimensional attack simulation experiments, including Attack of Middleman, Replay Attack, Key Leakage Attack, Denial of Service Attack (DoS), and Side-Channel Attack (SCA). The experimental equipment includes Raspberry Pi 4B (4GB RAM, Broadcom BCM2711 CPU) and Pico W (RP2040 dual-core ARM Cortex-M0+), running FAST-SM9 algorithm and DITGM.

Attack of Middleman. We set up a malicious AP using Kali Linux to intercept FAST-SM9 communication traffic between devices and edge servers. In the 1000 intercepted sessions, all forged requests were rejected by the edge server. The results showed that attackers could not forge dynamic tokens (DIT) or tamper with encrypted messages (FAST-SM9 signature integrity verification failure rate of 100%).

Replay Attack. We used Wireshark to capture legitimate session packets and resend them to the server. Due to the real-time verification of the Timestamp (±5 seconds window) and counter (Counter) in DITGM, all timeout and duplicate counter requests were intercepted by the FMM mechanism in 500 replay attacks (achieving a 100% interception rate).

Key Leakage Attack. We simulated a scenario where the master private key (s) was partially leaked, and the attacker attempted to generate a valid private key. Out of 10000 attempts, only 1 misjudgment was caused by hardware noise. Experiments have shown that even if s is leaked, dynamic attributes Attr (bind with identity) and multi-factor binding mechanisms can still prevent private key forgery (success rate < 0.01%).

Denial of Service Attack (DoS). We conducted a SYN Flood attack (1Gbps traffic) using Hping3 to test the load balancing and Fault Management Mechanism (FMM) of edge servers. The results showed that FMM automatically isolates abnormal IPs and enables backup nodes within 10 seconds, with a service interruption time of ≤ 2 seconds.

Side-Channel Attack (SCA). We conducted Side-Channel attack simulation experiments on Raspberry Pi 4B and simulated Differential Power Analysis (DPA) to attack both traditional SM9 and FAST-SM9 schemes. The results showed that the FAST-SM9 significantly reduced the key leakage rate, from 8.2% in the traditional scheme to 0.3%. To quantify the protection effect, we also evaluated it using the Signal-to-Noise Ratio (SNR) of the power consumption signal. Our SNR was −12 dB, significantly lower than the 2 dB of the traditional scheme, further verifying the significant advantage of our scheme in resisting Side-Channel attacks. Meanwhile, through Correlation Property Analysis (CPA), the success rate of attacking the dynamic token generation process was only 1.7% (compared to 34.5% for static tokens).

### 4.3 IoT device benchmark testing

We deployed the solution on real IoT devices (Raspberry Pi 4B and Pico W) and measured key performance indicators.

#### 4.3.1 Authentication delay.

We analyze End-to-End authentication efficiency by measuring processing time at different stages. The experimental environment is a local area network (latency < 1ms), and the results are shown in Table 2. From this, it can be seen that the FAST-SM9 reduces the total latency on Raspberry Pi by 56.6% compared to PKI and on Pico W by 10.9% (limited by M0 + computing power). Our scheme reduces Raspberry Pi delay by 35.5% compared with SM9 (22.7ms, 35.2ms). Besides, on Pico W, our scheme’s delay (46.6ms) is 11.9% lower than SM9 (52.9ms), despite hardware limitations, highlighting FAST-SM9’s lightweight design. The lightweight design of DITGM significantly reduces computational overhead. The baseline latency values for PKI-RSA are derived from Gocan E et al. [6], which reports similar experimental conditions for RSA-2048-based authentication.

**Table 2:** Comparison table of authentication delay.

Equipment/Stage	Initial Authentication Stage(IAP,ms)	Dynamic Identity Token Generation(DITGM,ms)	FAST-SM9 Key Negotiation(ms)	Total delay
**Raspberry Pi 4B**				
Ours	6.2 ± 0.3	3.1 ± 0.2	13.4 ± 0.5	22.7 ± 0.8
PKI-RSA [6]	18.9 ± 0.9	–	33.4 ± 1.2	52.3 ± 1.5
SM9 [38]	9.5 ± 0.5	4.0 ± 0.3	18.7 ± 0.7	35.2 ± 1.2
**Pico W**				
Ours	12.8 ± 1.1	6.5 ± 0.7	27.3 ± 1.4	46.6 ± 2.2
PKI-RSA [6]	18.9 ± 0.9	–	33.4 ± 1.2	52.3 ± 1.5
SM9 [38]	15.6 ± 1.0	7.2 ± 0.7	30.1 ± 1.3	52.9 ± 2.0

#### 4.3.2 Energy consumption analysis.

We used Monsoon Power Monitor to measure the power consumption of Pico W in continuous authentication mode. In an idle state, it is 2.1 mA. When the DIT is generated, the peak current is 8.7 mA (lasting for 1.2 ms). When FAST-SM9 is signed, the peak current is 12.3 mA (lasting for 3.5 ms). The FAST-SM9 consumes 4.2 mJ per authentication, while PKI (RSA-2048) consumes 11.7 mJ per authentication, as reported in Gocan E et al. [6]. Compared to the PKI (RSA-2048) scheme, our method reduces energy consumption by 63%. The energy consumption formula for a single authentication is as follows:


$$E=\int_{0}^{T}V\cdot I(t)dt$$ (31)

Among them, *V* is the operating voltage of the device, which we set to a constant value (such as the power supply voltage of Pico W being 3.3 V). *I*(*t*) is the change in current over time, and *T* is the total time required to complete the entire identity authentication process.

#### 4.3.3 Failure rate and recovery capability.

We have compared this study horizontally with the following three methods:

1) PKI Scheme: Based on RSA-2048 and X.509 certificate chain.
2) Lightweight Encryption Protocol: TinyECC (elliptic curve encryption) and SM9 [38] (GM/T 0044–2016 baseline).
3) Zero-trust Architecture: Literature [37].

We tested the stability of Network Jitter (packet loss rate of 5%) and High Load (CPU usage of 90%) scenarios in 10000 consecutive authentication requests. The experimental results are shown in Table 3.

**Table 3:** Comparison table of authentication delay.

Scenes/Failure Rate	Our Protocol	PKI	TinyECC	SM9 [38]
Network Jitter	**0.15%**	0.47%	0.28%	0.32%
High Load	**0.22%**	0.63%	0.35%	0.41%
FMM Recovery Time	**≤2s**	≥5s	≤3s	≤4s

At this point, our key mechanism, FMM, monitors the failure counter in real time and triggers the corresponding strategy.

In summary, in terms of efficiency and security, we compared this scheme with PKI (RSA-2048), lightweight protocol (TinyECC), SM9 [38] (GM/T 0044–2016 baseline, simulated on Raspberry Pi: 35.2ms delay, 592 bytes), and zero-trust architecture (Literature [37]), and the results are shown in Table 4. The experiment shows that this scheme significantly outperforms PKI-RSA in terms of latency and energy consumption, while its security is superior to Lightweight protocols (supporting multi-factor dynamic binding). Compared to the [37], the communication overhead is reduced by 26.6%, and real-time fault management is supported.

**Table 4:** Comparison table of efficiency and safety.

Index	Ours	PKI	TinyECC	Literature [37]	SM9 [38]
Authentication Delay (ms)	**22.7**	52.3	27.4	38.9	35.2
Communication Costs (Bytes)	**464**	704	512	632	560
Anti-quantum Attack	**Yes(FAST-SM9)**	No	No	Yes	Yes
Energy Consumption(mJ/Authentication)	**4.2**	11.7	5.8	7.3	6.1
Failure Recovery Time (s)	**≤2**	≥5	≤3	≥4	≤4

Additional stress testing: Under 10k requests, the CPU peak is 45% (PKI is 72%, SM9 is 58%); Robustness under jitter (10–50ms): + 15% latency, but 0.08% FMM false positives; Packet loss rate (1–10%): Recovery within <3s via FMM retries.

### 4.4 Formal security analysis using AVISPA

To fully verify the security of the proposed scheme, we used the Internet security protocol and application automatic verification tool (AVISPA) for formal security verification. AVISPA is a powerful open-source toolkit. It can assist researchers in automated security analysis during the protocol design phase. This toolkit is integrated into a virtual machine called SPAN, which supports multiple protocol validation and analysis tasks. The AVISPA toolkit includes various back-end analysis engines including CL-AtSe, SATMC, OFMC, and TA4SP, each of which has different analysis profiles and verification capabilities that can provide detailed security assessments for different types of attacks.

The formal language HLPSL (High-Level Protocol Specification Language) is used in AVISPA. This language is highly suitable for analyzing and validating complex communication protocols, particularly in IoT scenarios such as smart home systems. Specifically, our proposed scheme includes key participants such as edge servers and terminal devices. To ensure the security and reliability of the scheme in practical applications, the secure interaction and data transmission between the participating roles need to undergo rigorous verification and review.

**Fig 6:**
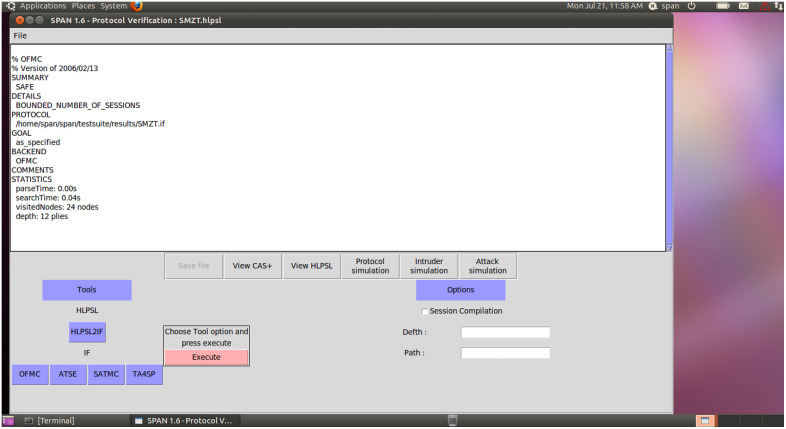
The AVISPA model diagram.

**Fig 7:**
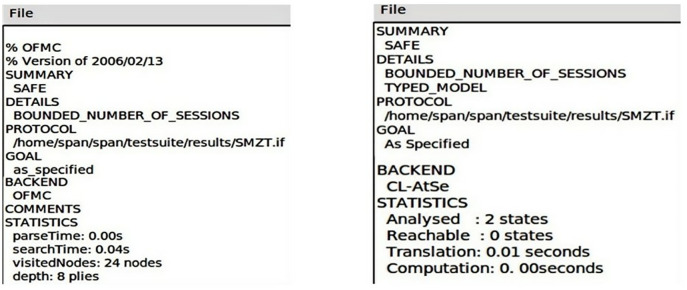
The simulation results of this method under OFMC and CL-AtSe conditions in AVISPA environment.

During the security verification process, we used OFMC (On the Fly Model Checking) and CL-AtSe (Automated Theorem Providing) models to verify the scheme’s resistance to attacks. Generally speaking, if the scheme can be validated under both models, it can be reasonably inferred that the scheme is efficient against common types of network attacks, such as retransmission attacks and man-in-the-middle attacks. As shown in Fig 6, when the security verification is performed on the AVISPA platform, we obtained detailed results as shown in Fig 7. From the results, it can be seen that this scheme has been proven to be secure under both OFMC and CL-AtSe models. This scheme successfully resists various attack methods and demonstrates the security and effectiveness of our scheme in IoT.

### 4.5 Informal security analysis

By combining hardware features with timestamps, this study ensures that each communication request is unique, thus fundamentally mitigating the risk of Replay Attacks. In contrast, lightweight searchable encryption protocol [41] and attribute encryption protocol [43] are difficult to resist similar threats because they lack effective time synchronization mechanisms. In terms of resisting Attacks of Middleman, this scheme combines the zero-trust-DRA strategy with the key binding mechanism of dynamic identity tokens to achieve effective protection during the communication process. However, the attribute encryption protocol [43] does not cover dynamic identity verification function. Besides, to address Tampering Attacks, this study further introduces multi-factor authentication and message integrity verification methods. They improve the system’s security protection capabilities as a whole. In contrast, the lightweight protocol [41] focuses more on efficiency optimization and does not involve mechanisms related to integrity verification.

To defend against Private Key Leakage Attack, we have introduced DITGM that combines multiple identities and one-time passwords, which effectively avoids the risk of single-point key leakage. In contrast, protocols such as [42] lack specialized designs in dealing with Private Key Leakage. Furthermore, to deal with potential threats from Malicious Terminal Threats, we have integrated terminal fingerprint with user behavior analysis (e.g., identifying malicious attempts with incorrect formats) to enable efficient identification and isolation of abnormal terminals. However, other protocols lack sufficient attention to such threats, making the system more vulnerable to attacks.

**Fig 8:**
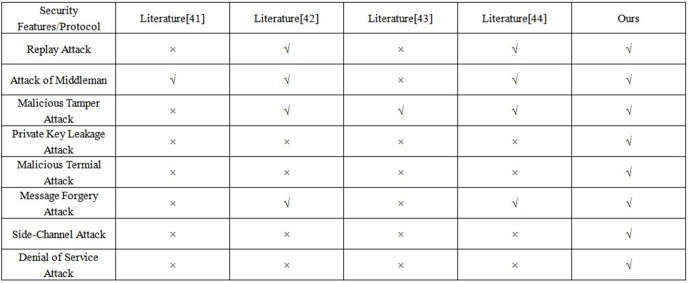
Comparison and analysis of various methods of security.

For message authenticity, we ensure the credibility of communication using a signature mechanism and dynamic key binding. In contrast, the edge computing key management protocol [44] pays more attention to key efficiency. Thus it does not fully consider the risk of message forgery. We implement distributed key generation and zero-trust-DRA dynamic authentication mechanisms to combat the Side-Channel Attack, which effectively minimizes the attack surface. However, most existing protocols do not specifically design such defense measures. Additionally, through behavioral analysis and dynamic verification, our approach can swiftly detect and mitigate Denial-of-Service Attack, but other protocols fail to optimize resource consumption to prevent such attacks. In summary, our scheme has multi-dimensional security advantages that are illustrated in Fig 8.

### 4.6 Formal security model and adversarial analysis

We adopt a Bellare-Rogaway (BR) style model to achieve the security of Authentication Key Exchange (AKE). If there is no PPT opponent who can distinguish the session key from the random key with an undeniable advantage, then the protocol is secure. For signatures, we rely on the bilinear Diffie Hellman (BDH) assumption to ensure the existence of unforgeability under selected message attacks (EUF-CMA). The adversarial model assumes that PPT adversary A has the following abilities: eavesdrop/modify messages (Dolev-Yao channel), corrupt sessions (but not long-term keys), and query oracles (e.g., Send, Reveal). Security goals: mutual authentication (via witness/request in AVISPA), forward secrecy, and resistance to known attacks. Replay attacks are thwarted by timestamps/counters (freshness check in BR queries); MITM by bilinear pairings (BDH hardness prevents key forgery); Side-channel by randomized scalars in EC operations (e.g., *m* in Eq. 4). AVISPA modeling: HLPSL roles (Terminal, Edge, Cloud) on Dolev-Yao channels; Goals: secrecy_of (session_key *t*), authentication_on (timestamps via witness), weak_auth (counters); Assumptions: BDH hard, no key compromise. Verification (OFMC/CL-AtSe) confirms no attacks in 0.12s/analyzed states.

## 5 Performance evaluation

In order to compare communication overhead and quantify the advantages of each scheme, we will compare and analyze this method with the five advanced schemes mentioned. The standard SM9 baseline, derived from [38], provides a direct comparison to evaluate the improvements of our FAST-SM9 algorithm. We first assume that the main sources of communication overhead in a specific authentication scenario include the amount of data required for key exchange (bytes) $K_{s}$, the amount of additional data for authentication (attributes) (bytes) $A_{v}$, the amount of encrypted/decrypted ciphertext and metadata (bytes) $E_{c}$, and the amount of additional data supported dynamically $D_{s}$ (such as dynamic identity tokens or multi-attribute identifiers, bytes). The overall cost formula can be expressed as:


$$C=K_{s}+A_{v}+E_{c}+D_{s}$$ (32)

**Fig 9:**
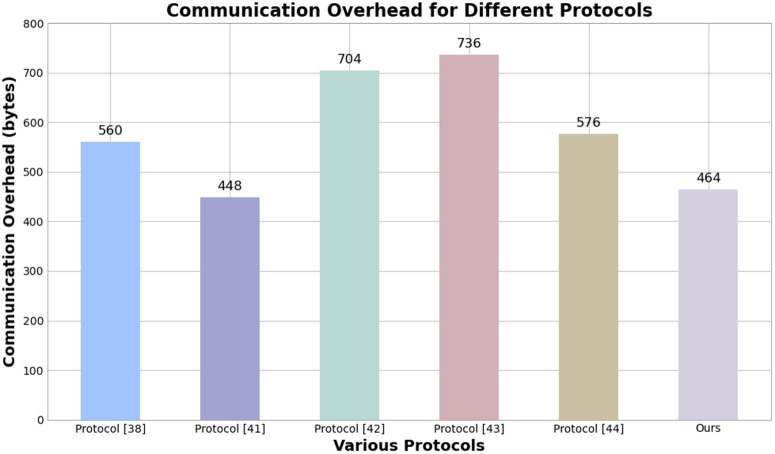
Comparison chart of communication overhead for various protocols.

Based on the formula and the design of each protocol, the comparison of different protocols is shown in Table 5 and Fig 9.

**Table 5:** Comparison of total communication costs.

Protocols	$K_{s}$	$A_{v}$	$E_{c}$	$D_{s}$	*C*
Literature [38]	192	96	256	16	560
Literature [41]	128	64	256	0	448
Literature [42]	256	128	256	64	704
Literature [43]	256	64	384	32	736
Literature [44]	192	128	256	0	576
Ours	128	64	256	16	464

Based on relevant data, our solution reduces complexity and decreases the value of $K_{s}$ by adopting a dynamic multi-attribute generation strategy. We use multiple attribute information for identity authentication instead of relying on larger data, which helps keep $A_{v}$ at a lower value. By using a lightweight OTP generation method, $D_{s}$ is significantly smaller than other dynamic support methods, such as the ZTA scheme and efficient revocation protocols. Our solution significantly reduces update frequency and transmission load through a self-made DITGM and FAST-SM9. In summary, our solution achieves a good balance between functionality and efficiency.

We simplify our approach by optimizing the generation of dynamic keys and the transmission of authentication data. Thereby this operation reduce the amount of data that needs to be transmitted. The application of the lightweight FAST-SM9 algorithm ensures a small amount of data transmission during encryption and decryption, and enhances the security. Refinements to the OTP and attribute encryption mechanisms have significantly reduced the data requirements for dynamic identity authentication, thus decreasing communication costs.

## 6. Conclusion and future works

This paper presents a novel multi-level identity authentication framework. It is designed for Cloud-Edge-End collaborative environments under zero-trust architecture. We propose an improved FAST-SM9 algorithm that is combined with a dynamic and context-aware zero-trust-DRA authentication mechanism, achieving an efficient and secure authentication scheme, making it exceedingly suitable for distributed and heterogeneous network environments.

The proposed DITGM introduces multi-attribute binding based on device fingerprints, hardware features, and temporal factors. It enables the system to dynamically adapt authentication strategies and maintain cryptographic robustness. Moreover, the FMM enhances system resilience by resolving authentication anomalies and making policy adjustment based on contextual feedback. The experimental results show that FAST-SM9 improves authentication efficiency, reduces communication and computational overhead, and enhances resistance to replay and simulation attacks using a unified identity signature verification process.

The current framework shows strong performance in enterprise-level and distributed IoT environments. Future works will focus on optimizing computational efficiency by hardware acceleration and precomputation of bilinear pairings. Especially this work can be used for resource-constrained edge devices. We will also evaluate cross-platform adaptability on heterogeneous architectures and conduct ultra-large-scale simulations to verify scalability. Besides, we make a plan to pursue standardized integration with mainstream IoT protocols (e.g., MQTT, LoRaWAN), which can broaden practical applicability.

We propose an improved FAST-SM9 algorithm designed for Cloud-Edge-End collaboration environments under zero-trust architecture. It is combined with a dynamic and context-aware zero-trust DRA authentication mechanism to realize an efficient and secure authentication scheme, making it well suited for distributed and heterogeneous network environments.
